# Heterogeneous antigenic properties of the porcine reproductive and respiratory syndrome virus nucleocapsid

**DOI:** 10.1186/s13567-016-0399-9

**Published:** 2016-11-21

**Authors:** Julie C. F. Rappe, Obdulio García-Nicolás, Franziska Flückiger, Barbara Thür, Martin A. Hofmann, Artur Summerfield, Nicolas Ruggli

**Affiliations:** 1 The Institute of Virology and Immunology IVI, Mittelhäusern, Switzerland; 2 Graduate School for Cellular and Biomedical Sciences, University of Bern, Bern, Switzerland; 3 Department of Infectious Diseases and Pathobiology, Vetsuisse Faculty, University of Bern, Bern, Switzerland; 4 Office for Consumer Protection, Canton Aargau, Obere Vorstadt 14, 5000 Aarau, Switzerland

## Abstract

Porcine reproductive and respiratory syndrome virus (PRRSV) is an arterivirus responsible for a widespread contagious disease of domestic pigs with high economic impact. Switzerland is one of the rare PRRSV-free countries in Europe, although sporadic outbreaks have occurred in the past. The PRRSV isolate IVI-1173 from the short outbreak in Switzerland in 2012 was entirely sequenced, and a functional full-length cDNA clone was constructed. Genetic and antigenic characterization of IVI-1173 revealed the importance of amino acid 90 of the nucleocapsid protein N as part of a conformational epitope. IVI-1173 was not detected by SDOW17, a monoclonal antibody against N widely used to detect PRRSV-infected cells. Substitution of alanine at position 90 of N [N(A_90_)] with a threonine [N(T_90_)] restored reactivity of vIVI1173-N(T_90_) to SDOW17 completely. The relevance of this amino acid for the conformational SDOW17 epitope of PRRSV N was further confirmed by the opposite substitution in a functional cDNA clone of the genotype 2 isolate RVB-581. Finally, N proteins from ten genotype 1 strains differing from threonine at position 90 were analysed for reactivity with SDOW17. N(A_90_) totally disrupted or severely affected the epitope in 7 out of 8 strains tested. Based on these findings, 225 genotype 1 strains were screened for the prevalence of N(A_90_). N(A_90_) is rare in classical subtype 1 and in subtype 3 strains, but is frequent in Russian subtype 1 (70%) and in subtype 2 (45%) isolates. In conclusion, this study highlights the variable antigenic properties of N among genotype 1 PRRSV strains.

**Electronic supplementary material:**

The online version of this article (doi:10.1186/s13567-016-0399-9) contains supplementary material, which is available to authorized users.

## Introduction

Porcine reproductive and respiratory syndrome (PRRS) is one of the economically most important viral disease of domestic pigs worldwide [1, 2]. The PRRS virus (PRRSV) has emerged in the late 1980s [3], with 2 genotypes, the European genotype 1 and the North American genotype 2 that have evolved independently in Europe and USA with approximately 60–70% nucleotide identity [4]. Genotype 1 is further subdivided in subtypes 1–3, while genotype 2 strains are classified into nine distinct lineages [5, 6]. In 2006, highly virulent genotype 2 strains emerged in China and Vietnam giving rise to outbreaks with severe symptoms of haemorrhagic fever [7]. More recently, genotype 1 strains with enhanced virulence were described in Eastern Europe [8]. Currently, only few countries are officially free from PRRSV, among which are Australia, New Zealand, Norway, Sweden and Switzerland [9–11].

PRRSV belongs to the genus *Arterivirus* of the family *Arteriviridae*, along with equine arteritis virus (EAV), lactate dehydrogenase elevating virus (LDV) of mice and simian haemorrhagic fever virus (SHFV) [12, 13]. According to the recent description of thirteen new arterivirus species, and in order to account for the clear divergence of the European and North American genotypes of PRRSV, it was proposed to reorganize the *Arteriviridae* family and to split PRRSV into two species, the *Suid 1 rodartevirus* and *Suid 2 rodartevirus*, grouped along with *Muroid rodartevirus* species in the genus *Rodartevirus* [14].

PRRSV is a small enveloped virus with a positive-sense, single-stranded RNA genome of approximately 15 kb carrying at least ten open reading frames (ORFs) [12]. ORF 1a and 1b encode 14 non-structural proteins processed proteolytically from the two polyproteins pp1a and pp1ab, and two additional proteins nsp2TF and nsp2N resulting from ribosomal frameshifts within the nsp2 gene [15, 16]. The remaining ORFs encode the structural proteins on a nested set of subgenomic messenger RNAs. ORF 2a, 2b and ORFs 3–5 encode the glycoprotein GP2, the small envelope protein E (ORF2b), the glycoproteins GP3 to 5 and the ORF5a protein. ORF6 and ORF7 are translated in the non-glycosylated membrane protein M and in the nucleocapsid protein N, respectively (reviewed in [17]).

The nucleocapsid protein N is a multifunctional, highly basic 15 kDa phosphoprotein whose major role is to associate with the viral genomic RNA to form the ribonucleocapsid [18–20]. N can self-associate through both, covalent and noncovalent interactions [21], and is found in the nucleus, nucleoli, and cytoplasm of infected cells [22]. It is the most abundant viral protein produced during PRRSV infection, inducing early antibody responses that do however not correlate with protection [23, 24]. N is one of the most conserved protein within isolates of the same genotype, although its heterogeneity within genotype 1 increased with the discovery of new Eastern European subtypes [5]. The N protein of genotype 1 and 2 PRRSV has a length of typically 128 and 123 amino acids, respectively, but its size varies between 124 and 132 amino acids for the subtypes 2 and 3 [5, 25–27]. N is the antigen of choice for PRRS serology and for monitoring infected cells during PRRSV isolation [28–30]. Epitopes of N were mapped accordingly, and broad reacting anti-PRRSV monoclonal antibodies (mAbs) against N were established [31–39]. SDOW17 is one of the most commonly used anti-N mAb which is considered to recognize N of nearly all European and North American PRRSV isolates except the PrimePac PRRS vaccine virus [31, 34, 40]. Accordingly, the SDOW17 mAb has been widely applied in immunohistochemistry [41, 42], immunofluorescence [43–45], virus titration [46], and flow cytometry [43, 47]. However, SDOW17 failed to detect the virus that caused the short PRRSV outbreak in Switzerland in 2012. This outbreak was due to import of contaminated semen from Germany and was resolved within a few months thanks to strict policy measures [48].

The present study was aimed at characterizing the isolate IVI-1173 recovered from an infected pig during this latter outbreak in Switzerland. The complete nucleotide sequence of IVI-1173 was determined and aligned with the currently known full-length PRRSV sequences, and a functional full-length cDNA clone was constructed. In particular, amino acid 90 of N was found to be critical for the recognition of N by the mAb SDOW17. Antigenic comparison of N from selected genotype 1 subtype 1, 2 and 3 PRRSV revealed the amino acid requirements of the SDOW17 epitope at position 90 of N.

## Materials and methods

### Cells

Porcine monocyte-derived macrophages (MDM) were generated as previously described [49]. Briefly, peripheral blood mononuclear cells were isolated from the blood of 6- to 18-month-old specific pathogen-free (SPF) Large White pigs from the breeding facility of The Institute of Virology and Immunology IVI in Switzerland (in compliance with the Swiss animal protection law, under licence number BE88/14 approved by the animal welfare committee of the canton of Bern, Switzerland) using ficoll-paque density centrifugation (1.077 g/L; GE Healthcare Life Sciences). Monocytes were then enriched by positive selection for CD172a with the antibody clone 74-22-15A (hybridoma kindly provided by Dr A. Saalmüller, Veterinary University of Vienna, Austria) using the magnetic cell sorting system (MACS) with LS columns (Miltenyi Biotec GmbH). The enriched monocytes were seeded at a density of 5 × 10^5^ cells per millilitre in Dulbecco’s modified Eagle’s medium (DMEM) without phenol red, supplemented with Glutamax (Life Technologies) and 10% heat-inactivated porcine serum from SPF pigs (IVI), and cultured at 39 °C with 5% CO_2_ for 72 h for differentiation to MDM which were then further maintained in this medium. BHK-21 cells were obtained from the German Cell Culture Collection (DSZM) and grown in Glasgow’s Minimum Essential Medium (Life Technologies) supplemented with 5% Tryptose Phosphate (Sigma-Aldrich) and 5% foetal bovine serum (Biowest). MARC-145 cells (ATCC, LGC Standards) were grown in DMEM (Life Technologies) supplemented with 10% foetal bovine serum. The BHK-21 and MARC-145 were maintained at 37 °C with 5% CO_2_.

### Viruses

PRRSV IVI-1173 was isolated in 2012 in the North-Eastern part of Switzerland from a secondary-infected pig following insemination of sows with contaminated boar semen imported from Germany [48]. The virus was passaged two times in porcine MDM. PRRSV RVB-581 was a kind gift from Martin Beer (Friedrich-Loeffler-Institut FLI, Greifswald-Insel Riems, Germany). The virus was collected in China in 2008 and isolated in porcine alveolar macrophages and MARC-145 cells at the FLI [50, 51]. PRRSV CReSA-2982 was isolated in MDM and kindly provided by Enric Mateu, Centre de Recerca en Sanitat Animal (CReSA, IRTA-UAB), Campus de la Universitat Autònoma de Barcelona, Bellaterra, Spain. All virus stocks were propagated in MDM. Cells were lysed by freezing and thawing at 50% cytopathic effect (CPE), clarified by centrifugation at 3000 × *g* and 4 °C for 10 min, and the supernatants were frozen at −70 °C. Lysates from MDM were used for mock infection controls. All strains were titrated in MDM by endpoint dilution using the immunoperoxidase monolayer assay (IPMA) with the PRRSV anti-N mAb SR30. Titers were expressed as 50% tissue culture infective dose/mL (TCID_50_/mL).

### Antibodies, IPMA, immunofluorescence (IF) and flow cytometry (FCM)

The anti-N mAbs SR30, SDOW17 (both from RTI, LLC) and 13E2 (kindly provided by Hans Nauwynck, University of Ghent, Belgium) were used for the detection of PRRSV by IPMA, IF and FCM. For IPMA, the cells were fixed and permeabilized with 80% acetone at room temperature, and incubated with the primary antibody and subsequently with goat anti-mouse IgG conjugated with horseradish peroxidase (Dako). Positive cells were visualized with AEC peroxidase substrate (0.05% [wt/vol] 3-amino-9-ethylcarbazole, 0.015% [vol/vol] H_2_O_2_, 0.05 M sodium acetate buffer, pH 5.5). For IF, the cells were fixed with paraformaldehyde 4% during 10 min at room temperature and permeabilized with 0.3% (wt/vol) saponin. The cells were then incubated with anti-PRRSV N antibody and with Alexa-488-conjugated goat anti-mouse IgG as secondary antibody (Thermo Fisher Scientific), both in presence of 0.3% (wt/vol) saponin. Nuclei were finally stained with DAPI (Sigma-Aldrich). Fluorescence microscopy was performed using an Axio Observer Z1 inverted microscope (Zeiss, Jena, Germany). For FCM, the cells were fixed with 4% paraformaldehyde at room temperature, and then incubated with the anti-N mAb and subsequently with goat anti-mouse IgG conjugated with Alexa-488, both in presence of 0.3% (wt/vol) saponin. FCM acquisition was done on a FACSCanto flow cytometer (Becton–Dickinson). Electronic gating based on the forward/side scatter plots was applied to identify living cells using the FlowJo V.9.7.6 software (Tree Star, Inc).

### Viral RNA extraction and nucleotide sequence analyses

For determining the complete PRRSV genome sequence of IVI-1173, serum from a pig infected during the Swiss outbreak of 2012 was passaged two times in MDM. For RVB-581, virus that had been passaged twice in MDM and once in MARC-145 at the Friedrich-Loeffler-Institut, Greifswald-Insel Riems, Germany, was used to infect pigs at the IVI (in compliance with the Swiss animal protection law, under licence number BE119/13 approved by the animal welfare committee of the canton of Bern, Switzerland), and serum at 6 days post infection was used for sequencing. Viral RNA was extracted from serum and MDM supernatant using TRIzol (Invitrogen). The final RNA pellet was resuspended in deionized water and stored at −70 °C in small aliquots. For IVI-1173 and RVB-581, 6 and 4 overlapping cDNA fragments respectively covering the full-length genome except the 5′ and 3′ terminal regions were synthesized by reverse transcription (RT) with Superscript III reverse transcriptase (Thermo Fisher Scientific) followed by PCR with Phusion Hot Start II DNA Polymerase (Thermo Fisher Scientific). The amplicons were inserted in the pJet1.2 plasmid using the CloneJET PCR Cloning Kit (Thermo Fisher Scientific). A total of 4 clones from 4 independent RT-PCR were sequenced for each fragment using the dideoxy-chain terminator sequencing chemistries of the BigDye Terminator v3.1 Cycle Sequencing Kit (Life Technologies) and an Applied Biosystems 3130 automated Genetic Analyzer (Applied Biosystems). The DNA was sequenced bi-directionally with forward and reverse primers. DNA sequences were assembled with the DNA baser software (version 3.5.3). The 5′ and 3′ termini of the viral genomes were determined using the 5′ and 3′ RACE System for Rapid Amplification of cDNA Ends (Invitrogen). Four clones from 4 independent RACE reactions each were sequenced in both directions. The consensus sequences of the complete viral genomes of IVI-1173 (accession number KX622783) and RVB-581 (accession number KX650082) were deposited to GenBank.

### Genome sequence alignments and phylogenetic analyses

Initial searches for nucleotide and amino acid sequence identities were carried out with NCBI’s Basic Local Alignment Search Tool for nucleotides (BLASTn) and proteins (BLASTp). Pairwise comparison and identity calculations were performed with the Clone Manager Professional Version 9 software (Scientific & Educational Software). Alignments and phylogenetic trees were generated with the Mega 6 software with bootstrap values based on 1000 replicates. All PRRSV genotype 1 strains with complete genome sequences deposited in GenBank at that time were used to construct the phylogenetic trees (see Additional file 1). For the trees based on ORF5 and ORF7, the 20 closest neighbours to IVI-1173—as determined by NCBI BLASTn and BLASTp—were added to all genotype 1 strains analysed in Ref. [5]. The genotype 2 PRRSV VR-2332 and RVB-581 were used as an out-group. Recombination analysis was performed with the recombination analysis tool (RAT) from John Innes Centre, Norwich Research Park, Norwich, UK, using the available full genomes of PRRSV-1 strains [52]. The GenBank references of all PRRSV strains used in this study are listed in the Additional file 1.

### Assembly of functional full-length cDNA clones of IVI-1173 and RVB-581

The complete viral genomes of IVI-1173 and RVB-581 were assembled under the control of the bacteriophage T7 RNA polymerase promoter in plasmids pACJR1 and pACJR2, respectively. These two vectors were derived from pACNR1180 [53] by replacing the 261 base pairs (bp) AatII to XhoI fragment with a 33 bp cassette containing the BglII and NotI restriction sites to generate pACJR1 or with a 32 bp cassette carrying the RsrII and NdeI restriction sites to obtain pACJR2, respectively. The complete genomes of IVI-1173 and RVB-581 were amplified by RT-PCR in 4 overlapping cDNA fragments each, directly from serum of the respective infected animals described above. Primers encoding the restriction endonuclease sites for cloning in pACJR1 or pACJR2 as well as the T7 polymerase promoter with 2 guanines at the transcription start site upstream of the first nucleotide of the genome in the 5′-terminal forward primer and a SwaI run-off restriction site in the 3′-terminal reverse primer were used to generate cDNA of the 5′ and 3′ ends of the genomes (the oligonucleotide sequences used can be obtained on request). The overlapping fragments of IVI-1173 and RVB-581 were assembled stepwise in pACJR1 and pACJR2 respectively, using the sites described above and unique restriction sites in the overlapping regions (details of the constructions can be obtained on request). All full-length plasmids were propagated in *Escherichia coli* XL-1 Blue and verified by nucleotide sequencing. All clones containing the consensus sequences determined above produced infectious virus upon transfection of cells with in vitro RNA transcripts (see below). The cDNA clones pIVI1173 #5 and pRVB581 #1 were used in the studies reported here.

### Transfection of in vitro transcripts and rescue of PRRSV

The plasmids pIVI1173 #5 and pRVB581 #1 were linearized with the restriction endonuclease SwaI at the 3′-terminal run-off site downstream of the polyA tail. Purified linearized DNA served as templates for in vitro transcription of capped RNA using the mMESSAGE mMACHINE Ultra T7 kit (Ambion) with m7G(5′)ppp(5′)G cap analog. The reaction mixture was treated with DNaseI to remove the template DNA and then purified with Illustra MicroSpin Columns S-400 HR (GE Healthcare Life Sciences). RNA was resuspended in water and stored at −70 °C in small aliquots. Size and integrity as well as concentration of the capped in vitro transcripts were determined by electrophoresis and measured with a Nanodrop 2000c spectrophotometer (Thermo Fisher Scientific), respectively. Virus was rescued by transfection of BHK-21 cells followed by infection of MDM. Briefly, BHK-21 cells were harvested, washed, and resuspended in PBS at a concentration of 20 × 10^6^ cells/mL. Ten μg of in vitro transcripts were added to 0.4 mL of cell suspension and electroporated with 2 pulses of 100 µs at 980 V with 1 s interval between the pulses using a ECM 830 Square Wave Electroporation System (BTX). After electroporation, 90% of the cells were diluted in cell growth medium and seeded in T75 flask. The remaining fraction of cells was seeded in 24-well plates to monitor PRRSV N protein expression by IPMA. At 24 h after transfection, the supernatant was collected after one freeze–thaw cycle, clarified by centrifugation, and used to infect porcine MDM. Rescue of infectious virus was confirmed by monitoring N protein expression by IPMA 48 h after infection of MDM and by virus titration in MDM.

### Construction of vIVI1173-N(T_90_) and vRVB581-N(A_90_)

The mutations encoding the amino acid substitutions A_90_T and T_90_A in N of pIVI1173 and pRVB581 respectively were generated using site-directed mutagenesis in short subcloned cDNA fragments using an overlapping extension PCR technique essentially as described before [54]. The mutated fragments were verified by nucleotide sequencing and transferred in the full-length plasmids. Details of the constructions can be obtained on request. The viruses vIVI1173-N(T90) and vRVB581-N(A90) were rescued as described above.

### Construction and transfection of PRRSV-ORF7 plasmids

The ORF7 cDNA cassettes of vIVI1173 and of the vIVI1173-N(T_90_) mutant were amplified from the respective functional full-length cDNA clones. The ORF7 cDNA cassette of 10 selected PRRSV strains with natural substitutions of threonine at position 90 of N were synthesized by GenScript (Piscataway, NJ, USA). All ORF7 cassettes were inserted in the NheI and NotI restriction endonuclease sites of pcDNA6/V5-His B and the resulting plasmids propagated in *Escherichia coli* XL-1 Blue. BHK-21 cells were transfected with 500 ng of each plasmid DNA purified with the NucleoBond Xtra Midiprep EF kit (Macherey–Nagel) using Lipofectamine 2000 (Invitrogen). Nucleocapsid N expression was monitored by FCM and IF.

## Results

### IVI-1173 is a genotype 1 subtype 1 PRRSV with atypical protein features

The PRRSV isolate IVI-1173 was recovered in porcine MDM from serum of an infected pig that was diagnosed PRRSV-positive with a quantitation cycle (Cq) value of 24 during a recent outbreak in Switzerland. A cytopathogenic effect became visible 24 h after infection, and nearly complete lysis of the cell monolayer was observed after 72 h. Surprisingly however, a PRRSV-specific staining of IVI-1173-infected MDM was observed only with the mAbs SR30 and 13E2 but not with mAb SDOW17 considered to detect nearly all PRRSV. This suggested atypical antigenic properties of N of IVI-1173.

In order to further characterize the IVI-1173 isolate, the complete nucleotide sequence was determined (GenBank accession number KX622783) and a functional full-length cDNA clone was constructed. The genome of IVI-1173 comprises 15065 nucleotides plus a polyA tail. The nucleotide identity of IVI-1173 with full-length genotype 1 sequences ranges from 80 to 89% while the virus shares only 61% nucleotide identity with the prototype genotype 2 strain VR-2332 and the highly pathogenic genotype 2 isolate RVB-581 (Table 1). The prototype genotype 1 strain Lelystad (LV) is the closest related strain overall, with 89% nucleotide identity based on the full-length nucleotide sequence. However, the nucleotide and amino acid identities of the individual genes of IVI-1173 and LV vary considerably from 81% to more than 97% identities depending on the gene or region compared (Table 2). Accordingly, separate nucleotide BLAST searches with the terminal untranslated regions (UTR) and with each ORF individually revealed genotype 1 strains other than LV that were more closely related to IVI-1173 (Table 3). In particular, based on ORF5, the closest strain is a German isolate from 2002 with 93% nucleotide and 95% amino acid identity, while the 5′ and 3′ UTR were the closest to Danish isolates. Despite this intra-genomic variability, the RAT recombination analysis tool software did not identify any potential recombination event.

**Table 1:** Nucleotide identity of IVI-1173 (15 065^a^) with representative PRRSV strains (in %)

	LV	Amervac	Euro-PRRSV	13V091	Lena	VR-2332	RVB-581
Genotype	1	1	1	1	1	2	2
Subtype	1	1	1	1	3	N.a.^d^	N.a.
Virulence	MV	Vaccine	Vaccine	HV	HV	MV	HV
Genome length^b^	15 098	15 098	15 047	15 020	15 001	15 411	15 320
Nt identity^c^ (%)	89	87	87	84	80	61	61

MV: moderately virulent; HV: highly virulent.

^a^IVI-1173 genome length without polyA tail used in the nucleotide sequence comparison.

^b^Genome length without polyA used in the nucleotide sequence comparison with IVI-1173.

^c^Nucleotide identity with IVI-1173 in %.

^d^Not applicable.

**Table 2:** Comparison of nucleotide (nt) and amino acid (aa) identities between IVI-1173 and LV

Sequence	Gene	% nt identity (length)	% aa identity (length)
Full length sequence	N.a.^a^	88.91% (15 065^b^/15 098^c^)	N.a.
5′ UTR	N.a.	96.38% (221)	N.a.
ORF1a	N.a.	87.76% (7191)	89.11% (2396)
	Nsp1	86.32% (1155)	87.27% (385)
	Nsp2	85.33% (2583)	81.30% (861)
	Nsp3	90.68% (1341)	95.30% (447)
	Nsp4	89.82% (609)	95.57% (203)
	Nsp5	91.76% (510)	95.88% (170)
	Nsp6	93.75% (48)	93.75% (16)
	Nsp7a	86.13% (447)	95.97% (149)
	Nsp7b	90.83% (360)	94.17% (120)
	Nsp8	88.15% (135)	100% (45)
ORF1b	N.a.	90.19% (4392)	95.76% (1463)
	Nsp9	90.59% (1935)	96.28% (645)
	Nsp10	90.20% (1326)	95.70% (442)
	Nsp11	89.88% (672)	95.54% (224)
	Nsp12	88.89% (459)	94.08% (152)
ORF2a	GP2	92.4% (750)	93.98% (249)
ORF2b	E	95.31% (213)	97.15% (70)
ORF3	GP3	81.83% (777/798)	82.26% (258/265)
ORF4	GP4	84.24% (519/552)	83.61% (172/183)
ORF5	GP5	89.77% (606)	90.55% (201)
ORF5a	5a	96.97% (132)	95.35% (43)
ORF6	M	91.76% (522)	95.38% (173)
ORF7	N	94.83% (387)	94.53% (128)
3′ UTR	N.a.	94.74% (114)	N.a.

^a^Not applicable.

^b^Genome length of IVI-1173 without polyA tail.

^c^Genome length of LV without polyA tail.

**Table 3:** Nucleotide and protein BLAST analysis of the IVI-1173 sequence

Genome region	Nucleotide BLAST					Protein BLAST				
	Closest strain	Accession number	Origin	Year	Nt^a^	Closest strain	Accession number	Origin	Year	AA^b^
Full length	LV	M96262	Netherlands	1993	89	N.a.^c^	N.a.	N.a.	N.a.	N.a.
5′ UTR	DK-1992	KC862566	Denmark	1992	97	N.a.	N.a.	N.a.	N.a.	N.a.
ORF 1a	LV	M96262	Netherlands	1993	88	LV	M96262	Netherlands	1993	89
ORF 1b	CReSA-3266	JF276434	Germany	1996	90	LV	JF276434	Spain	2011	96
ORF 2	LV	M96262	Netherlands	1993	92	SD-01-07	AAR32995	USA	2003	96
ORF 2b	DK-2012-01-05-2	KC862574	Denmark	2012	96	14432/2011	ALH24924	Hungary	2011	100
ORF 3	E38	KT033457	South Korea	2007	89	Sp-28a	AEM00458	Spain	2011	86
ORF 4	Sp-16a	JF730930	Spain	2011	85	Sp-29a	AEM00498	Spain	2011	87
ORF 5	BH 95/10-08	JN651737	Germany	2002	93	BH 95/10-08	JN651737	Germany	2002	95
ORF 5a	K09-1351	JQ656126	Korea	2009	99	13V117	ALO24147	Belgium	2013	98
ORF 6	BH 95/10-08	JN651718	Germany	2002	93	469-05	AFH96401	UK	2005	96
ORF 7	Be1	L77914	UK	1995	96	LA2	AGT28447	Austria	2008	96
3′ UTR	DK-2003-7-2	KC862572	Denmark	2003	98	N.a.	N.a.	N.a.	N.a.	N.a.

^a^Percentage of nucleotides identity.

^b^Percentage of amino acid identity.

^c^Not applicable.

**Figure 1:**
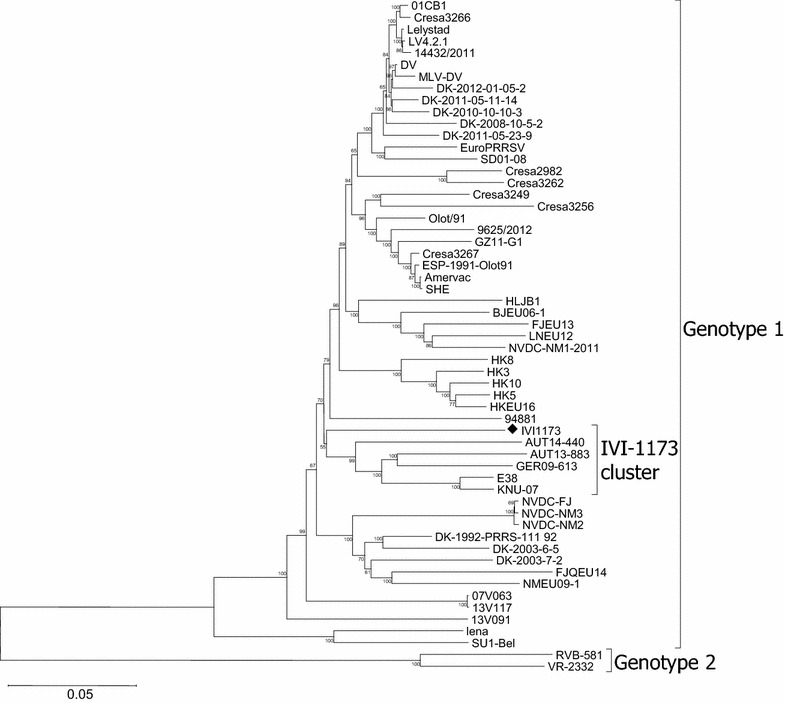
**Phylogenetic analysis of the IVI-1173 genome in the context of 55 complete genotype 1 PRRSV genomes.** The tree was constructed by the Neighbour Joining method with the numbers at the nodes representing bootstrap values in % of 1000 replicates. The evolutionary distances were computed using the p-distance method and are given in the number of base differences per site for a total of 14 008 positions in the final dataset. IVI-1173 (diamond) and its cluster are indicated. The evolutionary analyses were conducted in MEGA6.

**Figure 2:**
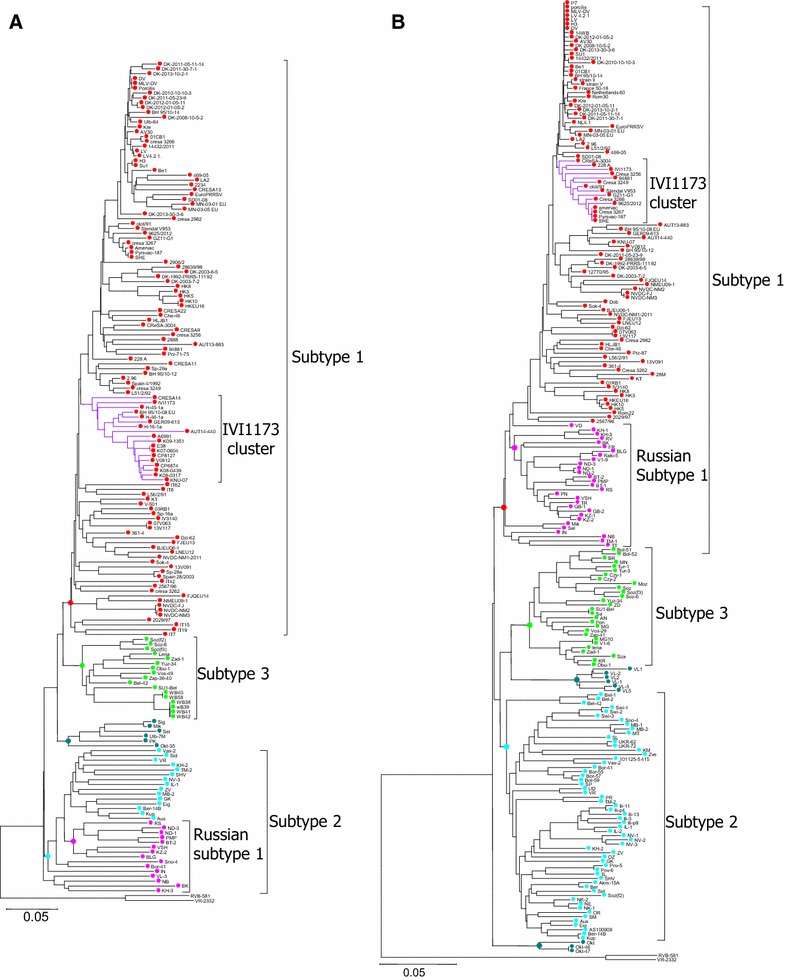
**IVI-1173 clusters within genotype 1 subtype 1 PRRSV.** The trees were constructed by the Neighbor Joining method and represent a phylogenetic analysis using 174 ORF5 (**A**) and 225 ORF7 (**B**) nucleotide sequences from GenBank. The subtypes are represented with different colours.

The different clustering depending on the genome region was confirmed with phylogenetic analyses. A phylogenetic tree including 55 full-length PRRSV genome sequences showed that IVI-1173 belongs clearly to genotype 1, clustering with two Austrian, one German and two South Korean strains. However, this association was supported by a bootstrap value of 55 only (Figure 1). A phylogenetic tree based on 174 ORF5 sequences confirmed the grouping of IVI-1173 within the classical subtype 1 PRRSV and the close relationship to strains from Austria, Spain, Germany and South Korea (Figure 2A). The affiliation of IVI-1173 to subtype 1 PRRSV was also confirmed with 225 ORF7 sequences, but the closest related subtype 1 isolates were from USA, Hungary, Portugal, Denmark, China, Germany and Spain. Here, IVI-1173 clustered also with two vaccine strains, Amervac and Pyrsvac-187 (Figure 2B).

**Figure 3:**
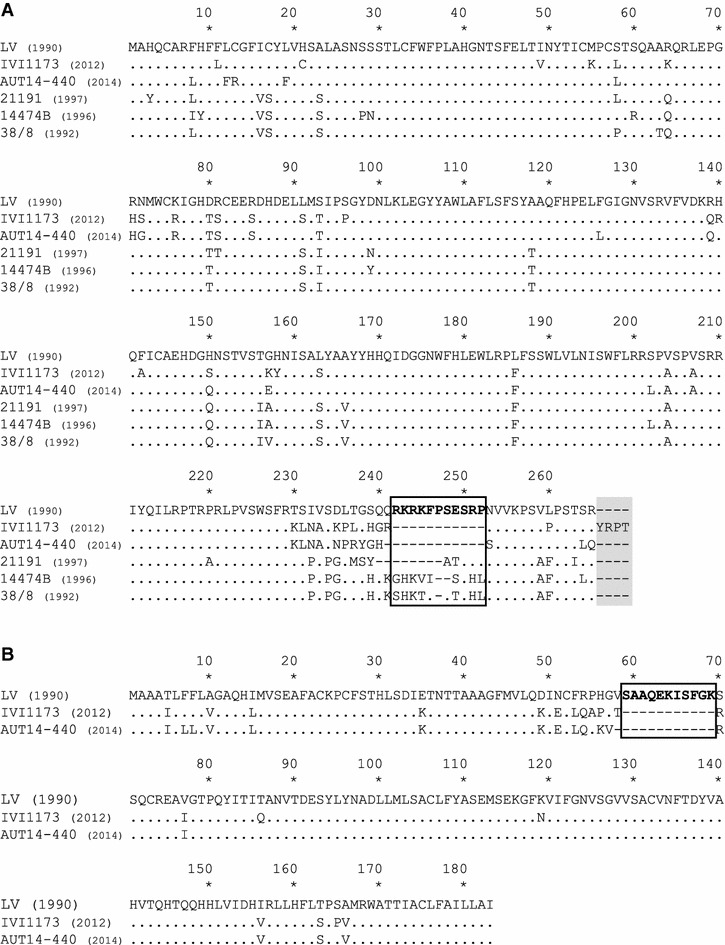
**Alignment of the GP3 and GP4 amino acid sequences of selected PRRSV genotype 1 isolates.** In comparison with LV, the amino acid deletions of different lengths in the overlapping region of GP3 (**A**) and GP4 (**B**) of selected PRRSV genotype 1 isolates are boxed, and the 4 additional amino acids at the C-terminus of GP3 are shaded.

**Figure 4:**
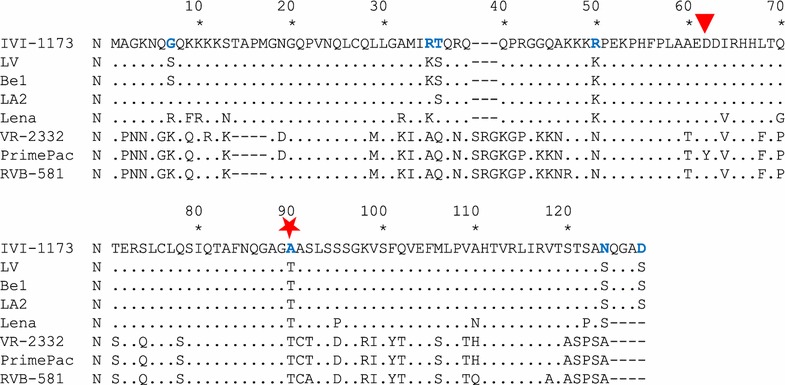
**Alignment of the N amino acid sequences of IVI-1173 and of representative PRRSV strains.** The 7 amino acid differences between the genotype 1 prototype strain LV and IVI-1173 are shown in blue bold letters. The red star highlights the substitution of the conserved threonine at position 90 of N with an alanine in IVI-1173. Be1 and LA2 (genotype 1) are the closest to IVI-1173 at the nucleotide and amino acid level of N. VR-2332 is the prototype genotype 2 PRRSV. Lena and RVB-581 are highly virulent genotype 1 subtype 3 and genotype 2 strains, respectively. PrimePac (genotype 2) is the only strain for which the lack of N detection by SDOW17 was reported, which is due to substitution of a conserved aspartic acid at position 62 with a tyrosine (red triangle).

An important feature was a 33 nucleotide deletion in the IVI-1173 genome where the ORF3 and ORF4 mRNAs overlap, leading to an 11 amino acid deletion in each of the two proteins when compared with the GP3 and GP4 from LV, near the C-terminal end of GP3 and downstream of amino acid 58 of GP4, respectively (Figures 3A and B). Moreover, GP3 of IVI-1173 has 4 additional amino acids at the carboxy-terminal end (Figure 3A). The N protein of LV and IVI-1173 differ by 7 amino acids (Figure 4). At position 62 of N (highlighted with a triangle in Figure 4), where PrimePac carries a tyrosine responsible for the lack of reactivity of N with SDOW17 [40], there is a conserved aspartic acid in IVI-1173. Therefore, this residue could not explain the lack of reactivity of IVI-1173 with the SDOW17 mAb. The most prominent difference found in IVI-1173 compared with other PRRSV was an alanine at position 90 of N [N(A_90_)] instead of a conserved threonine at this position (represented by a star in Figure 4).

### The amino acid at position 90 of N is critical for the conformational epitope recognized by the mAb SDOW17

**Figure 5:**
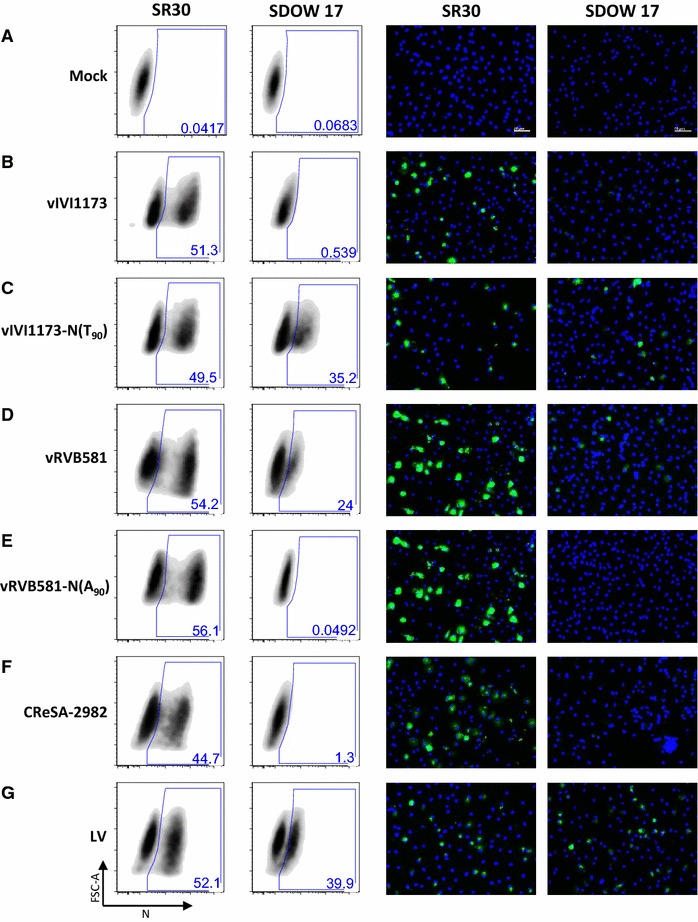
**The amino acid at position 90 of PRRSV N is crucial for the epitope recognized by SDOW17.** MDM were mock infected (**A**) or infected with vIVI1173 or the mutant vIVI1173-N(T_90_) (**B**, **C**), with vRVB581 or the mutant vRVB581-N(A_90_) (**D**, **E**) or with CReSA-2982 or LV as controls (**F**, **G**). After 18 h, N detection by flow cytometry (left two panels) and by immunofluorescence staining of the monolayers (right two panels) was performed with mAbs SR30 and SDOW17.

Since position 62 of N is obviously not involved in the lack of reactivity of IVI-1173 with the SDOW17 mAb (see above), we investigated the potential role of position 90 for this epitope by replacing the N(A_90_) in the cDNA-derived vIVI1173 with the conserved threonine [N(T_90_)]. As expected, cells infected with vIVI1173 were clearly positive for N expression with mAb SR30 whereas no signal was obtained with SDOW17 (Figure 5B). The threonine at position 90 restored detection of vIVI1173-N(T_90_)-infected cells with SDOW17, whereas the signal obtained with SR30 remained unchanged (Figure 5C), suggesting that threonine at position 90 belongs to the conformational epitope recognized by SDOW17. This was confirmed by disrupting the putative SDOW17 epitope at position 90 of N in the backbone of the genotype 2 strain RVB-581. To this end, the N(T_90_) was substituted with an alanine in the cDNA-derived vRVB581. MDM infected with vRVB581 were clearly positive for N expression by immunodetection with both, SR30 and SDOW17 (Figure 5D) whereas infection with the mutant vRVB581-N(A_90_) was detected with SR30 but not with SDOW17 (Figure 5E). Moreover, MDM infected with the isolate CReSA-2982 harbouring an alanine at position 90 of N did not react with SDOW17 neither, while they were positive with SR30 (Figure 5F). Altogether, these data demonstrate that position 90 of N is part of the conformational epitope recognized by SDOW17 and suggest the requirement of a threonine at this position.

### Field strains detected by the mAb SDOW17 harbour typically threonine or serine at position 90 of N

**Figure 6:**
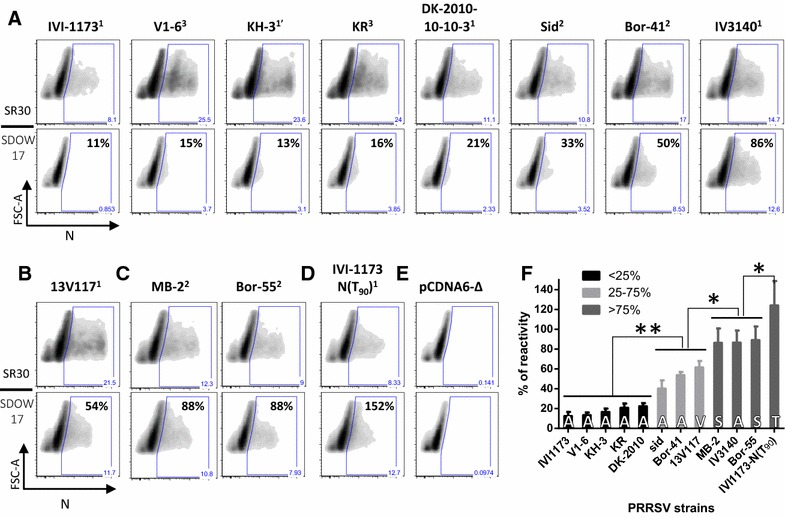
**Comparative reactivity of N from various PRRSV strains with the mAb SDOW17.** The PRRSV N genes from genotype 1 PRRSV strains of different subtypes (^1^ = classical subtype 1, ^1′^ = Russian subtype 1, ^2^ = subtype 2, ^3^ = subtype 3) with an alanine (**A**), a valine (**B**), a serine (**C**), or a threonine (**D**) at position 90 were cloned in pcDNA6/V5-His and expressed in BHK-21 cells. N expression was monitored 24 h after lipofection by flow cytometry using the SDOW17 and SR30 mAbs. The gate of N-positive cells was determined according to the density plot obtained with the cells transfected with the empty plasmid, pCDNA6-Δ (**E**). The percentage of SDOW17 reactivity compared with the SR30 reactivity is indicated for each SDOW17 panel (**A**–**D**). The mean values from 2 independent experiments performed in triplicate are plotted, with error bars showing the standard deviation and with the amino acid at position 90 shown at the bottom of each bar (**F**). The N proteins of the different strains were grouped according to the ratio of SDOW17 to SR30 detection (% of reactivity). Black, light grey and dark grey bars represent the strains with less than 25%, between 25 and 75%, and more than 75% N detection by SDOW17 versus SR30, respectively. The differences between groups were assessed statistically by the Mann–Whitney U test (*p < 0.05; **p < 0.01).

Based on the results obtained above, we determined whether the reactivity of N with SDOW17 can be predicted from the sequence at position 90 of natural PRRSV isolates and whether other residues than threonine are tolerated at this position. To this end, the N proteins from ten field strains harbouring natural substitutions of threonine at position 90 were expressed transiently in BHK-21 cells transfected with plasmids for eukaryotic expression of the different N genes. Detection of N by flow cytometry (Figure 6) and immunofluorescence (Additional file 2, panel A) using SDOW17 and SR30 in parallel showed that the ratios of the SDOW17 to SR30 reactivity were significantly reduced in all N carrying an alanine at position 90, except for the Korean strain IV3140 (Figures 6A and F; Additional file 2). The detection of the 13V117 isolate carrying a valine at this position was only partially impaired (Figures 6B and F; Additional file 2) while the two viruses with a serine at position 90 were detected nearly as efficiently as vIVI1173-N(T_90_) (Figures 6C, D and F; Additional file 2).

### Substitutions of threonine at position 90 of N are highly prevalent in Russian subtype 1 and in subtype 2 PRRSV strains

**Figure 7:**
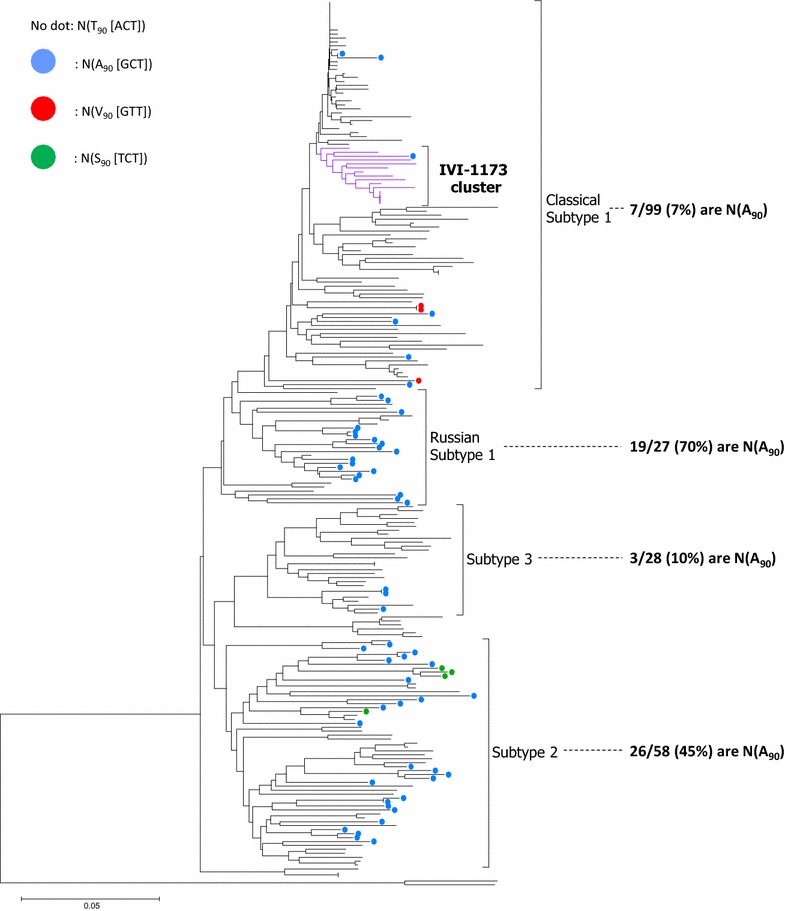
**Prevalence of N(A**_**90**_ **) in genotype 1 PRRSV.** PRRSV strains carrying an amino acid other than a threonine at position 90 of N are represented by colored dots in a phylogenetic tree based on 225 ORF7 sequences, with blue for alanine, red for valine and green for serine. The fraction (and percentage) of strains harboring an alanine at position 90 are indicated for each subtype.

Considering the requirements of amino acid 90 of N for the detection of virus infection by SDOW17, we investigated the prevalence of an alanine at position 90 of N [N(A_90_)] in a wide panel of N sequences from PRRSV genotype 1 strains available from GenBank (Figure 7; Additional file 1). It appears that N(A_90_) is found in 70% of the Russian subtype 1 strains and in 45% of the subtype 2 strains [5]. Valine and serine at this position are less frequent. These findings are certainly to be considered in PRRSV isolation and detection procedures of these subtypes in particular.

## Discussion

During a short PRRSV outbreak in Switzerland in 2012 [48], it was noticed that the virus had unusual antigenic properties of N, as infected cells failed to react with the mAb SDOW17 commonly used in PRRSV diagnostics. Complete genomic sequence analysis of the isolate IVI-1173 recovered from a viremic pig revealed a genotype 1 subtype 1 PRRSV with atypical protein features of GP3, GP4 and N. Reverse genetics was used to demonstrate that the lack of IVI-1173 N detection by SDOW17 was due to an alanine at position 90 of N instead of a threonine present in most classical PRRSV subtype 1 isolates. Importantly, these data highlight an antigenic feature of N with unexpected high prevalence in Russian subtype 1 and in subtype 2 PRRSV.

The IVI-1173 isolate clustered with the genotype 1 subtype 1 strains, irrespectively of whether the phylogenetic analyses employed ORF5 or ORF7 sequences used typically for PRRSV genotyping [5]. This was in line with the general observation that subtyping of genotype 1 leads to similar results with the two ORFs [18, 50], with the exception of Russian strains for which the subtyping is incongruent [5, 6]. These latter strains group within subtype 1 in phylogenetic trees based on ORF7 and within subtype 2 in trees based on ORF5. Interestingly, we found a high proportion of strains with N(A_90_) among both, the Russian subtype 1 and the subtype 2 isolates, compared to the classical subtype 1 strains. Thus our data highlight an antigenic characteristic shared essentially by the Russian subtype 1 and the subtype 2 isolates (Figure 7), which in this case is consistent with subtyping based on ORF5 showing a close relationship of these two subtypes (Figure 2), as opposed to the phylogeny based on ORF7 [6].

An interesting observation was the 11 amino acid deletion in each of the two glycoproteins GP3 and GP4. This deletion lies within a neutralizing domain of GP3 and GP4 identified in LV [55, 56]. Interestingly, a 12 amino acid deletion at this same position was reported for the isolate AUT14-440 closely related to IVI-1173 and isolated very recently in Austria [57] (Figures 3A and B). The deletion may be the consequence of selective pressure exerted by neutralizing antibodies. This has already been postulated with strains from the early 1990s that exhibited shorter amino acid deletions within this epitope [58] (Figure 3A).

Another interesting feature of IVI-1173 was the lack of reactivity of N with SDOW17. We used reverse genetics to demonstrate the involvement of amino acid 90 of N in the conformational epitope recognized by this mAb. Reactivity of N with SDOW17 was restored completely by substituting alanine with a threonine at this position in the IVI-1173 backbone. Disruption of the epitope in the RVB-581 backbone by the opposite substitution demonstrated definitively the importance of this residue for detection of PRRSV-infected cells by SDOW17. Several mAbs were found to detect common antigenic regions of genotype 1 and 2 PRRSV. One common domain was found between amino acids 50 and 66 of N using the Olot/91 (European) and Québec 807/94 (North American) isolates [32]. Overall, at least 4–5 antigenic domains were mapped in N using a panel of mAbs [29, 33, 34, 56] (Additional file 2, panel B, Additional file 3). While most mAbs reacted with linear peptides, one group of mAbs including SDOW17 recognized a discontinuous epitope in N of the two genotypes. For the genotype 1 prototype LV, SDOW17 recognized amino acids 51–67 and 80–90 [33] (Additional file 2, panel B, yellow shading). For the PA-8 strain, a genotype 2 related to VR-2332, SDOW17 binding was mapped to residues 30–52 and 112–123 [34] (Additional file 2, panel B, dark blue boxes). The SR30 epitope was localized between residues 69 and 123 in PA-8 [34] (Additional file 2, panel B, light blue box). Both, the SR30 and SDOW17 mAbs do not detect N in Western blots [34]. The epitope recognized by mAb 13E2 could not be identified by pepscan analysis [56]. Interestingly, SDOW17 was long regarded as a nearly universal antibody for PRRSV detection, except for the PrimePac vaccine strain that was considered as one of the rare viruses not detected by this mAb [40]. The failure of SDOW17 to detect the PrimePac virus was mapped to a tyrosine that had replaced a conserved aspartic acid at position 62 (Figure 4; Additional file 2, panel B). With amino acid 90, we identified a critical residue from the second domain of the conformational epitope of SDOW17. According to the crystal structure of N [59], this residue is located within a beta-strand. N forms dimers in which the two antigenic regions 51–67 and 80–90 are located close to each other [59]. Tentative in silico modelling of the effect of an alanine substitution at this position using the Garnier-Osguthorpe-Robson method suggests a disruption of the beta strand towards a more helical structure (not shown).

An important finding resulting from the present data demonstrating the role of N(T_90_) for the SDOW17 epitope is the unexpected high prevalence of an alanine at this position [N(A_90_)] in the subtype 1 strains from Russia and in subtype 2 isolates. Most of the PRRSV partial sequence data from GenBank are determined from RT-PCR without virus isolation. Therefore, little is known on the antigenic properties of the proteins from the viruses sequenced. We used synthetic cDNA fragments to express a selection of ten PRRSV N proteins from different subtypes, all carrying a predicted disrupted SDOW17 epitope. Apart from the South Korean isolate IV3140, all N proteins carrying the N(A_90_) residue were not or only weakly detected by SDOW17. Serine could substitute for threonine without nearly any loss of function. However, the picture was not black and white, suggesting intermediate affinities with alanine or valine at position 90 depending probably on compensatory changes elsewhere in the conformational epitope. By comparing the sequences of N of the Bor-41, Sid and IV3140 strains that are detected by SDOW17 at different degrees despite the presence of N(A_90_) with the sequences of the non-detected strains carrying N(A_90_), one may predict potential compensatory mutations in the region encompassing residues 34–50 and 121–124 of the Bor-41, Sid and IV3140 strains (see Additional file 2, panel B). These two regions were previously identified as part of the epitope detected by SDOW17 in genotype II strains [34] (Additional file 2, panel B, Additional file 3). Moreover, modelisation of the IVI-1173 and IV3140 N proteins with the protein homology/analogy recognition engine version 2.0 (Phyre^2^, [60]) showed a helix structure in N of IV3140 between residues 123-125, which is not found in N of IVI-1173 (Additional file 4). However the confidence values for this latter structure were low. Eventually, reverse genetics studies are required to identify the residues that allow SDOW17 binding to IV3140 N despite N(A_90_). The relatively high prevalence of N(A_90_) may suggest a functional relevance of this epitope in vivo. Since N is not exposed on the virion surface, a selective pressure driving amino acid substitutions in N may be related to immunological selection to evade T cell responses in particular porcine genetic backgrounds [5, 61].

Due to the high genetic diversity of PRRSV, only few commercial antibodies are available. SDOW17 has been widely used as nearly universal anti-PRRSV mAb. In addition, new diagnostic approaches relying on the universal properties of monoclonal antibodies have been developed using SDOW17 as a prototype [62]. From a general point of view, our study shows that antigen detection based on a single mAb must be considered with care. This was emphasised in a recent review claiming that lack of knowledge on the properties and affinities of antibodies are a major cause of non-reproducibility in research and diagnostics [63].

## Additional files

**Additional file 1.** **GenBank references of PRRSV strains used in the present study.** The strain name, country of origin, collection date, and the GenBank accession numbers are listed for each sequence used in the present study. The names of the strains with complete genome sequences are shaded in grey. Consistent with the color code used in Figure 7, the GenBank accession numbers of the ORF7 sequences containing an alanine (blue), a valine (red) or a serine (green) at position 90 of the N protein are highlighted.

**Additional file 2.** **Comparative reactivity of N from various PRRSV strains with mAb SDOW17 and predicted antigenic regions of N.** **A** In analogy to the data of Figure 6, the reactivity of the mAbs SDOW17 and SR30 was determined against the different PRRSV N proteins expressed transiently in BHK-21 cells using IF. The secondary antibody was alexa-488-conjugated (green) and the cell nuclei were counterstained with DAPI (blue). The N proteins of the different strains were grouped according to the flow cytometry results of Figure 6. Black, light grey and dark grey shading highlight the strains with less than 25%, between 25 and 75%, and more than 75% N detection by SDOW17 versus SR30, respectively. **B** All ORF7 amino acid sequences of the strains used in the experiment of panel A and in Figure 6 were aligned and compared to the IVI-1173 sequence using the Clone Manager software. The sequence names are highlighted in black, light grey and dark gray according to the grouping described above. The predicted SDOW17 epitope region mapped for LV is highlighted in yellow [33]. The SDOW17 epitope regions mapped with PA-8 are delimited by dark blue rectangles [34]. The region harboring the SR30 epitope in PA-8 is delimited by a light blue rectangle [34]. LV, VR-2332, PA-8 and PrimePac were included as reference strains. The amino acid differences between IV3140 and IVI-1173 are shown in bold red.

**Additional file 3.** **Selected epitopes of PRRSV N protein recorded in the immune epitope database (IEDB).** Table compiling IEDB data [64] for selected epitopes of the PRRSV N protein.

**Additional file 4.** **Predicted secondary structure of the IVI-1173 and IV-3140** **N protein.** The secondary structure of the N protein of IVI-1173 and IV3140 was predicted using the Phyre2 software from the Phyre2 web portal for protein modeling, prediction and analysis [60].

## Abbreviations

aa: amino acids
bp: base pair
Cq: quantitative cycle
FCM: flow cytometry
IF: immunofluorescence
LV: Lelystad virus
mAb: monoclonal antibody
MDM: monocytes-derived-macrophages
N: nucleocapsid protein
N(A_90_): alanine at position 90 of N
N(T_90_): threonine at position 90 of N
N(S_90_): serine at position 90 of N
N(V_90_): valine at position 90 of N
PRRS: porcine reproductive and respiratory syndrome
PRRSV: porcine reproductive and respiratory syndrome virus
wt: wild type
