# Supplementary material for: Heterogeneous antigenic properties of the porcine reproductive and respiratory syndrome virus nucleocapsid
Source: Vet Res. 2016 Nov 21;47:117. doi: 10.1186/s13567-016-0399-9 (PMC5118883; doi:10.1186/s13567-016-0399-9)
Supplement: Supplementary file 1 — Additional file 1. GenBank references of PRRSV strains used in the present study. The strain name, country of origin, collection date, and the GenBank accession numbers are listed for each sequence used in the present study. The names of the strains with complete genome sequences are shaded in grey. Consistent with the color code used in Figure 7, the GenBank accession numbers of the ORF7 sequences containing an alanine (blue), a valine (red) or a serine (green) at position 90 of the N protein are highlighted. [file 13567_2016_399_MOESM1_ESM.pdf]

Strain (grey = complete genome)	Country of origin	Collection date	Accession number ORF5	Accession number ORF7
2.96	Denmark?	<1997	AY035925	Z92532
2234	Austria		AY875858	--
2888	Austria		AY875855	--
94881	USA	2006	KT988004	KT988004
01CB1	Thailand	<2006	DQ864705	DQ864705
01RB1	Thailand	2001	--	AY796316
03RB1	Thailand		AY297124	--
07V063	Belgium	2007	GU737264	GU737264
101125-5_415	Belarus	2010	--	KC714005
13V091	Belgium	2013	KT159248	KT159248
13V117	Belgium	2013	ALO24147	ALO24147
14432/2011	Hungary	2011	ALH24924	ALH24924
14WB	Slovakia	2011	--	KF205271
2029/97	Italy	1997	AY035930	AY035973
228 A	Denmark?	<2001	AY035909	AY035954
2567/96	Italy	1996	AY035932	AY035976
28/2003	Spain		DQ345755	--
28639/98	Denmark	1998	AY035912	AY035957
28M	Slovakia	2009	--	JX046540
2906/2	Austria		AY875862	--
361-4	Denmark	1994	AY035915	AY035960
469-05	U.K.	2005	JN86241	JF896255
9625/2012	Hungary	2012	KJ415276	KJ415276
A6991	Korea	2012	KF360875	--
Akm-15A	Lithuania	2008	--	KC714006
Amervac PRRS	Spain	vaccine	GU067771	GU067771
AN	Belarus	2001	--	EU071252
AS100908	Lithuania	2010	--	KC714007
Aus	Lithuania	2000	DQ324667	AF438362
AUT13-883	Austria	2013	KT326148	KT326148
AUT14-440	Austria	2014	KT334375	KT334375
AV30	?	<1997	AY035901	Z92708
Be1	U. K.	1993	JN862385	L77914
Bel-1	Belarus	2010	--	KC714008
Bel-2	Belarus	2010	--	KC714009
Bel-42	Belarus	2004	DQ324669	DQ324699
Ber	Lithuania	2007	--	KC714010
Ber-14B	Lithuania	2005	KC714025	KC714011
BH_95/10-08_EU	Germany	2002	JN651737	JN651699
BH_95/10-12	Germany	2006	JN651738	JN651700
BH_95/10-14	Germany	2008	JN651740	JN651702
BJEU06-1	China	2006	GU047344	GU047344
BK	Russian Federation	2006	EU071231	EU071258
BLG	Russian Federation	2006	EU071232	EU071259
Bol-51	Belarus		--	KC714012
Bol-52	Belarus		--	KC714013
Bor-41	Belarus	2004	DQ324671	DQ324701
Bor-55	Belarus	2009	--	KC714014

Bor-57	Belarus	2009	--	KC714015
Bor-59	Belarus	2009	--	KC714016
BR	Belarus	2001	--	EU071254
BT-1	Russian Federation	2000	--	EU071302
BT-2	Russian Federation	2006	EU071247	EU071303
Che-46	Poland	2005	DQ324673	DQ324703
CP6874	South Korea		EF031042	--
CP8127	South Korea	<2006	EF031043	--
CRESA 11	Spain		DQ009626	--
CRESA 13	Spain		DQ009637	--
CRESA 14	Spain	<2006	DQ009638	--
CRESA 22	Spain		DQ009645	--
Cresa 2982	spain	2005	JF276430	JF276430
Cresa 3249	spain	2005	JF276433	JF276433
Cresa 3256	spain	2005	JF276432	JF276432
Cresa 3262	spain	1992	JF276431	JF276431
Cresa 3266	Germany	1996	JF276434	JF276434
Cresa 3267	Portugal	2006	JF276435	JF276435
CRESA 9	Spain		DQ009634	--
CReSA-3004	spain	1994	GQ451669	GQ451692
Czy-1	Belarus	2009	--	KC714017
Czy-2	Belarus	2009	--	KC713968
DK-1992-PRRS-111_92	Denmark	1992	KC862566	KC862566
DK-2003-6-5	Denmark	2003	KC862571	KC862571
DK-2003-7-2	Denmark	2003	KC862572	KC862572
DK-2008-10-5-2	Denmark	2008	KC862573	KC862573
DK-2010-10-10-3	Denmark	2010	KC862568	KC862568
DK-2011-05-11-14	Denmark	2011	KC862567	KC862567
DK-2011-23-9	Denmark	2011	KC862569	KC862569
DK-2011-30-7-1	Denmark	2011	KC862513	KC862544
DK-2012-01-05-11	Denmark	2012	KC862518	KC862548
DK-2012-01-05-2	Denmark	2012	KC862574	KC862574
DK-2013-10-2-1	Denmark	2013	KC862524	KC862551
DK-2013-30-3-6	Denmark	2013	KC862525	KC862552
Dob	Poland	2007	--	EU071225
DV	Netherlands	1996	KF991509	KF991509
DZ	Russian Federation	2000	--	EU071276
Dzi-62	Poland	2005	DQ324675	DQ324705
E38	South korea	2007	KT033457	KT033457
Eig	Lithuania	2006	KC714026	KC713968
EuroPRRS	USA		AY366525	AY366525
FJEU13	China	<2015	KP860912	KP860912
FJQEU14	China	<2015	KP860913	KP860913
FR	Russian Federation	2000	--	EU071260
France 50-18	France?	<2000	--	AF297102
GB-1	Russian Federation	1997	--	EU071261
GB-2	Russian Federation	2000	--	EU071262
GER09-613	Germany	2009	KT344816	KT344816
GK	Russian Federation	2005	EU071251	EU071308
GZ11-G1	China	2011	KF001144	KF001144

H-16-1a	Germany	2004	FJ705402	--
H3	U.K.	<1997	JN862375	L77916
H-45-1a	Germany	2005	FJ705427	--
H-46-1a	Germany	2004	FJ705429	--
HK10	Hong kong	2004	KF287131	KF287131
HK3	Hong kong	2003	KF287129	KF287129
HK5	Hong kong	2004	KF287130	KF287130
HK8	Hong kong	2004	KF287128	KF287128
HKEU16	Hong kong	<2007	EU076704	EU076704
HLJB1	China	2014	KT224385	KT224385
IL-1	Russian Federation	1997	EU071240	EU071277
IL-2	Russian Federation	1999	--	EU071278
Ili-11	Russian Federation	2009	--	KC713971
Ili-13	Russian Federation	2009	--	KC713972
Ili-3	Russian Federation	2009	--	KC713970
Ili-p4	Russian Federation	2009	--	KC713973
Ili-p8	Russian Federation	2009	--	KC713974
IN	Russian Federation	2005	EU071237	EU071270
IT15	Italy	2004	AY739971	--
IT19	Italy	2002	AY739975	--
IT42	Italy	2003	AY739998	--
IT62	Italy	2003	AY743932	--
IT7	Italy	2004	AY739963	--
IT8	Italy	2004	AY739964	--
IV3140	South Korea		DQ355821	DQ355822
IVI1173	Switzerland	2012	KX622783	KX622783
K07-0604	South Korea	2007	JQ656013	--
K08-0317	South Korea	2008	JQ656028	--
K08-0439	South Korea	2008	JQ656050	--
K09-1351	Korea	2009	JQ656126	--
KH-1	Russian Federation	2004	--	EU071266
KH-2	Russian Federation	2005	EU071234	EU071267
KH-3	Russian Federation	2005	EU071235	EU071268
KM	Russian Federation	1999	--	EU071265
KNU-07	South Korea	2007	FJ349261	FJ349261
KR	Russian Federation	2000	--	EU071271
Kre	Lithuania		KC714028	KC713975
KT	Czech Republic	2006	EU071226	EU071224
Kup	Lithuania	2007	KC714029	KC713976
KZ-1	Russian Federation	1999	--	EU071274
KZ-2	Russian Federation	2004	EU071239	EU071275
L51/2/92	?	<1997	AY035937	Z92531
L56/2/91	Spain	1991	AY035935	AY035979
LA2	Austria	2008	KC522617	AGT28447
Lelystad	The Netherlands	1991	M96262	M96262
Lena	Belarus	2007	JF802085	JF802085
LNEU12	China	2012	KM196101	KM196101
LV 4.2.1	Netherlands	<2004	AY588319	AY588319
MB-1	Russian Federation	2004	--	EU071272
MB-2	Russian Federation	2005	EU071238	EU071273

MG	Belarus	1999	--	EU071255
MG10	Russian Federation	2010	--	KC713977
Mik	Latvia	2008	KC714030	KC713978
MLV-DV	netherlands	<1999	KJ127878	KJ127878
MN	Belarus	1999	--	EU071253
MN-03-01_EU	USA?	<2004	AY749377	AY749378
MN-03-05_EU	USA?	<2004	AY749381	AY749382
Moz	Belarus	2010	--	KC713979
MT	Russian Federation	2009	--	KC713980
NB	Russian Federation	2006	EU071241	EU071280
ND-1	Russian Federation	1998	EU071248	EU071304
ND-2	Russian Federation	1999	--	EU071305
ND-3	Russian Federation	2006	EU071249	EU071306
NE	Russian Federation	1999	--	EU071290
netherlands 60	Netherlands	<2000	--	AF297100
NK-1	Russian Federation	1999	--	EU071291
NK-2	Russian Federation	2004	--	EU071292
NL4.1	?	<1997	--	Z92534
NMEU09-1	China	2009	GU047345	GU047345
NV-1	Russian Federation	1996	--	EU071281
NV-2	Russian Federation	2004	--	EU071282
NV-3	Russian Federation	2006	EU071242	EU071283
NVDC-FJ	China	2011	KC492506	KC492506
NVDC-NM1-2011	China	2011	JX187609	JX187609
NVDC-NM2	China	2011	KC492504	KC492504
NVDC-NM3	China	2011	KC492505	KC492505
Obu-1	Belarus	2005	DQ324676	DQ324707
Okt	Belarus	2010	--	KC713981
Okt-35	Belarus	2004	DQ324677	--
Okt-46	Belarus	2004	--	DQ324708
Okt-47	Belarus	2005	--	ABC74843
Olot/91	spain	1991	KF203132	KF203132
OR	Russian Federation	2000	--	EU071284
P7	Czech Republic	2009	--	KF134439
PK	Belarus	2002	EU071229	--
PMP	Russian Federation	2006	EU071243	EU071285
PN	Russian Federation	2000	--	EU071286
Pon	Belarus	2010	--	KC713982
Porcilis PRRS	The Netherlands	vaccine	DQ324678	DQ324710
Pov-5	Belarus	2010	--	KC713983
Pov-6	Belarus	2010	--	KC713984
PR	Russian Federation	1998	--	EU071287
Prz	Poland	2005	DQ324680	DQ324711
Pyrsvac-187	Spain	vaccine	--	DQ324712
Raki-5	Belarus	2010	--	KC713985
Rom22	Romania		--	JX134062
Rom30	Romania	2011	--	JX134070
RS	Russian Federation	2005	EU071230	EU071257
RV	Russian Federation	2003	--	EU071263
RVB-581	China	2006	KX650082	KX650082

SD01-08	USA	<2006	DQ489311	DQ489311
Sel	Latvia	2009	KC714032	KC713986
SHE	China	<2008	GQ461593	GQ461593
SHV	Russian Federation	2006	EU071236	EU071269
Sid	Lithuania	2000	DQ324682	AF438363
Sig	Latvia	2009	KC714033	--
SL	Russian Federation	2010	--	KC713987
SM	Russian Federation	1998	--	EU071288
Sno-4	Belarus	2004	DQ324683	DQ324713
Sok-4	Poland	2004	DQ324684	DQ324715
Soz	Belarus	2009	--	KC713988
Soz(f2)	Belarus	2006	EU071227	EU071222
Soz(f3)	Belarus	2006	EU071228	EU071223
Soz-6	Belarus	2004	DQ324686	DQ324719
SP	Russian Federation	1999	--	EU071289
Sp-16a	Spain	2011	JF730969	--
Sp-28a	spain	2011	JF730975	--
Sp-29a	Spain	2011	JF730976	--
Spain 4/1992	spain	1992	DQ345731	--
Stendal_V953	Germany	1996	JN651728	JN651690
strain II	?	<1997	--	Z92526
strain V	?	<1997	--	Z92706
SU1	U.K.	1992	JN862384	JN862244
Su1-bel	Belarus	2010	KP889243	KP889243
Svi	Belarus	2010	--	KC713989
Swi-1	Belarus	2009	--	KC713990
Swi-2	Belarus	2009	--	KC713991
Swi-3	Belarus	2009	--	KC713992
Sza	Belarus	2010	--	KC713993
TL	Russian Federation	1999	--	EU071296
TM-1	Russian Federation	2000	--	EU071293
TM-2	Russian Federation	2005	EU071244	EU071294
TR	Russian Federation	2009	--	KC713994
TT	Russian Federation	1998	--	EU071295
Tur-1	Belarus	2008	--	KC713995
Tur-3	Belarus	2008	--	KC713996
UD	Russian Federation	2001	--	EU071298
UKR-62	Ukraine	2008	--	KC713997
UKR-72	Ukraine	2008	--	KC713998
Ulb-64	Latvia	2009	KC714036	--
Ulb-7M	Latvia	2009	KC714035	--
V0812	Korea	2008	GQ847591	GQ847617
V1-6	Russian Federation	2010	--	KC713999
V1-9	Russian Federation	2010	--	KC714000
V-501	Czech Republic	1996	AF253531	--
Vas-2	Belarus	2005	DQ324689	DQ324722
VD	Russian Federation	1997	--	EU071279
VL1	Russian Federation	2009	--	KC714001
VL-1	Russian Federation	2001	--	EU071299
VL2	Russian Federation	2010	--	KC714002

VL-2	Russian Federation	2004	--	EU071300
VL-3	Russian Federation	2006	EU071246	EU071301
VL5	Russian Federation	2009	--	KC714003
Vos-29	Belarus	2004	--	DQ324725
Vos-49	Belarus	2004	DQ324690	--
VR	Russian Federation	2005	EU071233	EU071264
VR2332	USA	1990	EF536003	EF536003
VSH	Russian Federation	2005	EU071250	EU071307
WB38	Lithuania		KC714037	--
WB39	Lithuania		KC714038	--
WB40	Lithuania		KC714039	--
WB41	Lithuania		KC714040	--
WB42	Lithuania		KC714041	--
WB58	Lithuania		KC714042	--
Yuz-34	Belarus	2004	DQ324692	DQ324727
Zad-1	Belarus	2004	DQ324694	DQ324729
Zap-36-40	Belarus	2004	DQ324696	--
Zap-41	Belarus	2004	--	DQ324734
ZD	Belarus	2000	--	EU071256
ZV	Russian Federation	2004	EU071245	EU071297
Zve	Belarus	2009	--	KC714004
