# Supplementary figures and images for: Heterogeneous antigenic properties of the porcine reproductive and respiratory syndrome virus nucleocapsid
Source: Vet Res. 2016 Nov 21;47:117. doi: 10.1186/s13567-016-0399-9 (PMC5118883; doi:10.1186/s13567-016-0399-9)

**A**

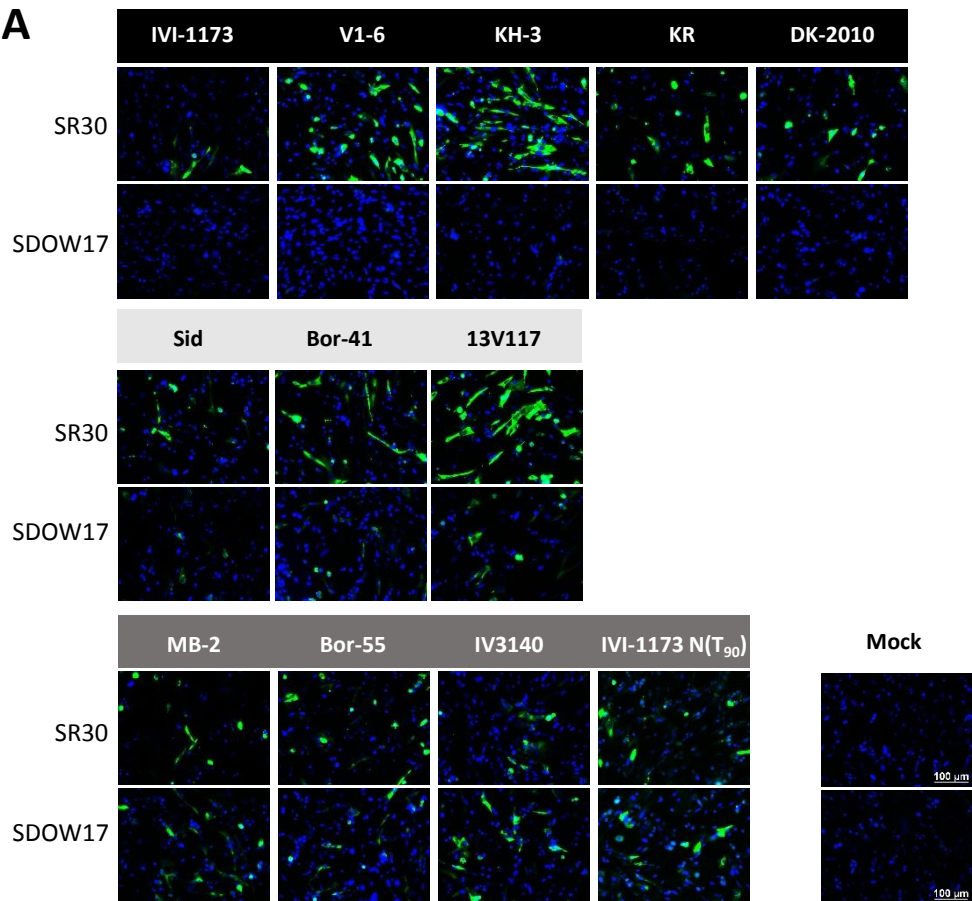

**B**

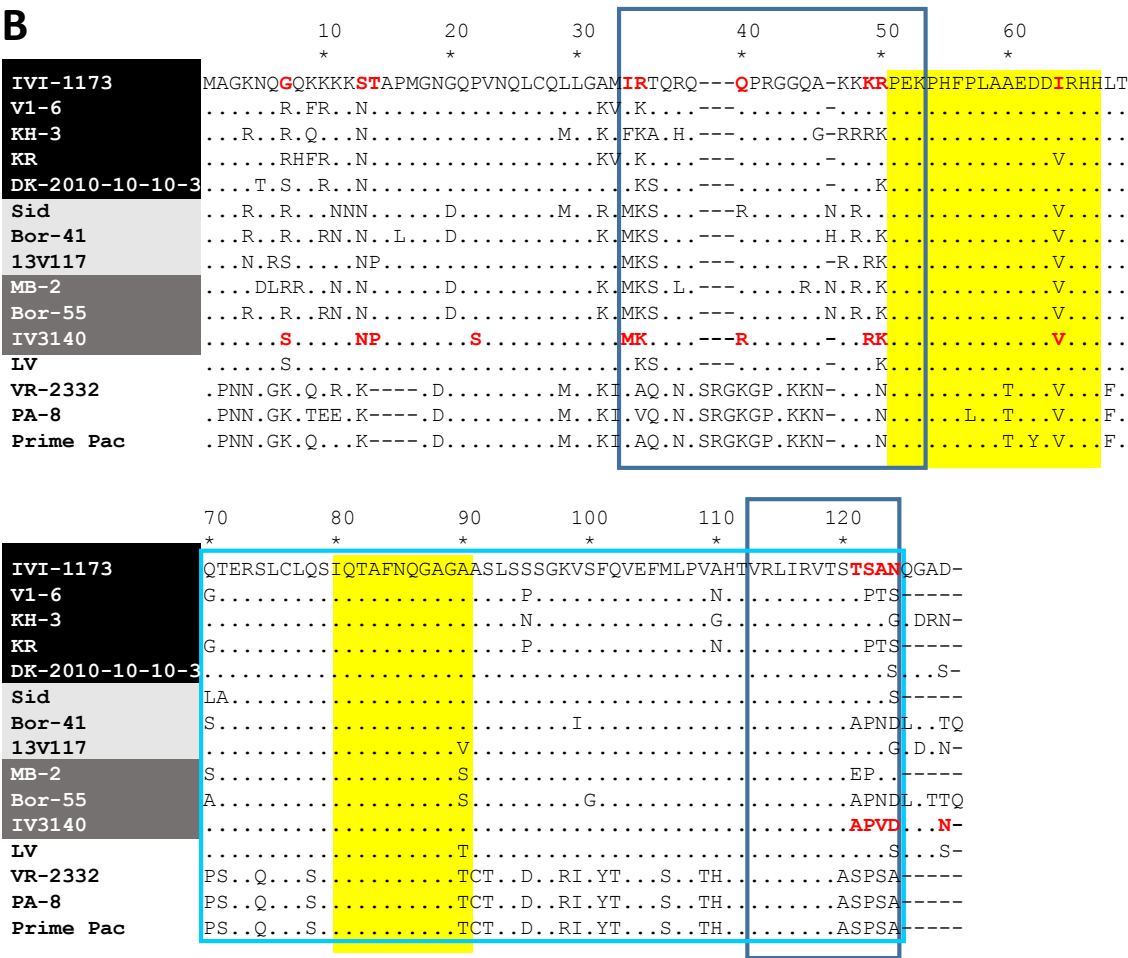

Supplement: Supplementary file 2 — Additional file 2. Comparative reactivity of N from various PRRSV strains with mAb SDOW17 and predicted antigenic regions of N. A In analogy to the data of Figure 6, the reactivity of the mAbs SDOW17 and SR30 was determined against the different PRRSV N proteins expressed transiently in BHK-21 cells using IF. The secondary antibody was alexa-488-conjugated (green) and the cell nuclei were counterstained with DAPI (blue). The N proteins of the different strains were grouped according to the flow cytometry results of Figure 6. Black, light grey and dark grey shading highlight the strains with less than 25%, between 25 and 75%, and more than 75% N detection by SDOW17 versus SR30, respectively. B All ORF7 amino acid sequences of the strains used in the experiment of panel A and in Figure 6 were aligned and compared to the IVI-1173 sequence using the Clone Manager software. The sequence names are highlighted in black, light grey and dark gray according to the grouping described above. The predicted SDOW17 epitope region mapped for LV is highlighted in yellow [33]. The SDOW17 epitope regions mapped with PA-8 are delimited by dark blue rectangles [34]. The region harboring the SR30 epitope in PA-8 is delimited by a light blue rectangle [34]. LV, VR-2332, PA-8 and PrimePac were included as reference strains. The amino acid differences between IV3140 and IVI-1173 are shown in bold red. [file 13567_2016_399_MOESM2_ESM.pdf]

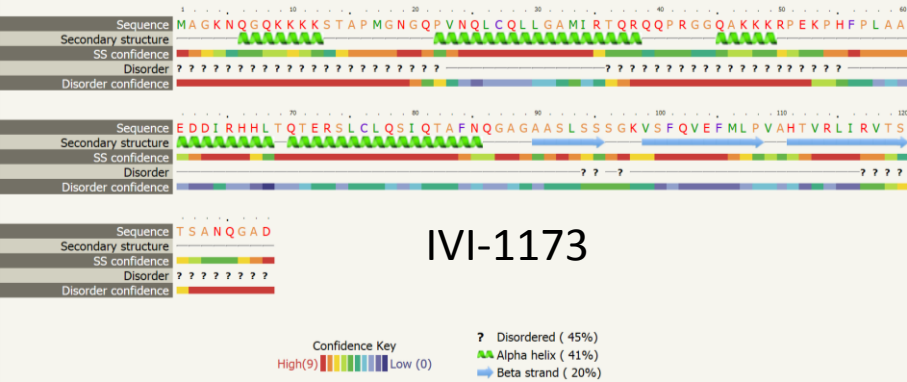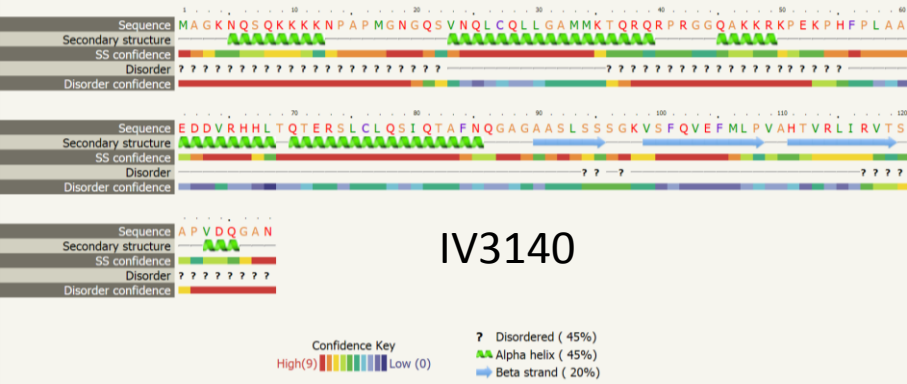

Supplement: Supplementary file 4 — Additional file 4. Predicted secondary structure of the IVI-1173 and IV-3140 N protein. The secondary structure of the N protein of IVI-1173 and IV3140 was predicted using the Phyre2 software from the Phyre2 web portal for protein modeling, prediction and analysis [60]. [file 13567_2016_399_MOESM4_ESM.pdf]
