# Supplementary material for: Heterogeneous antigenic properties of the porcine reproductive and respiratory syndrome virus nucleocapsid
Source: Vet Res. 2016 Nov 21;47:117. doi: 10.1186/s13567-016-0399-9 (PMC5118883; doi:10.1186/s13567-016-0399-9)
Supplement: Supplementary file 3 — Additional file 3. Selected epitopes of PRRSV N protein recorded in the immune epitope database (IEDB). Table compiling IEDB data [64] for selected epitopes of the PRRSV N protein. [file 13567_2016_399_MOESM3_ESM.pdf]

Additional file 3. Epitopes of the nucleocapsid protein N of PRRSV

Strain	Position[a)]	Epitope[b)]	References
LV	2-12	AGKNQ**S**QKKKK	[33]
		AGKNQ**G**QKKKK	
	25-30	QLCQLL	
		QLCQLL	
	40-46	QPRGGQA	
		QPRGGQA	
	51-67 + 80-90♠	PEKPHFPLAAEDDIRHH + IQTAFNQGAG**T**	
		PEKPHFPLAAEDDIRHH + IQTAFNQGAG**A**	
	25-34	QLCQLLGAMI	[29]
		QLCQLLGAMI	
	31-40	GAMI**KS**QRQQ	
		GAMI**RT**QRQQ	
	46-55	AKKK**K**PEKPH	
		AKKK**R**PEKPH	
	44-71	GQAKKK**K**PEKPHFPLAAEDDIRHHLTQT	
		GQAKKK**R**PEKPHFPLAAEDDIRHHLTQT	
	50-93	**K**PEKPHFPLAAEDDIRHHLTQTERSLCLQSIQTAFNQGAG**T**ASL	
		**R**PEKPHFPLAAEDDIRHHLTQTERSLCLQSIQTAFNQGAG**A**ASL	
	50-58♦	**K**PEKPHFPL	[39]
		**R**PEKPHFPL	
	64-72♦	IRHHLTQTE	
		IRHHLTQTE	
	105-113♦	FMLPVAHTV	
		FMLPVAHTV	
	113-121♦	VRLIRVTST	
		VRLIRVTST	
VR-2332	21-48	QLCQMLGKIIAQQNQSRGKGPGKKNKKK	[29]
	26-34	LGKIIAQQN	
	30-57	IAQQNQSRGKGPGKKNKKKNPEKPHFPL	
	36-46	SRGKGPGKKNK	
	42-50	GKKNKKKNP	
	43-70	KKNKKKNPEKPHFPLATEDDVRHHFTPS	
	48-57	KNPEKPHFPL	
	63-92	VRHHFTPSERQLCLSSIQTAFNQGAGTCTL	
	103-123	EFSLPTHHTVRLIRVTASPSA	
PA-8	30-52♠	IVQQNQSRGKGPGKKNKKKNPEK	[34]
	37-57	RGKGPGKKNKKKNPEKPHFLL	
	52-69	KPHFLLATEDDVRHHFTP	
	112-123♠	VRLIRVTASPSA	
Jxwn06	49-57♦	**N**PEKPHFPL	[38]
		**R**PEKPHFPL	
	104-120♦	F**S**LP**TQ**HTVRLIR**A**T**AS**	
		F**M**LP**VA**HTVRLIR**V**T**ST**	

a) SDOW17 epitopes are highlighted by the symbol ♠ whereas T cell epitopes are indicated by ♦

b) For the epitopes of LV and Jxwn06, the second row shows the corresponding sequence of IVI-1173 with the differences highlighted with red bold characters.
